# "The Hospital" Nursing Mirror

**Published:** 1897-03-20

**Authors:** 


"The Hospital" Nursing Mirror.

Being the Nursing Section of "The Hospital."

[Contributions for this Section of "The Hospital" should be addressed to the Editor, The Hospital, 28 & 29, Southampton Street, Strand, London, W.C., and should have the word "Nursing" plainly written in left-hand top corner of the envelope.]

### News from the Nursing World.

### THE QUEEN'S REIGN.

A meeting was held in Dublin on the 15th inst., at which it was resolved to open a City and County Dublin Commemoration Fund, in celebration of the sixtieth year of the Queen's reign, "to be administered in accordance with the rules of the Queen's Jubilee Institute for the Training of Nurses in Ireland." A number of subscriptions were acknowledged, and it was announced that the Lord Lieutenant would subscribe £500.—At Truro it has been unanimously decided to form a "Cornwall Association of Nurses," to be affiliated with the Q.V.J.I. At a meeting held on Monday, under the presidency of Lord Mount-Edgcumbe, a provisional committee was appointed to draw up the details of the scheme.—At Cheltenham a fund is to be raised for the endowment and development of the Cheltenham Victoria Nursing Home, originally started as a memorial of Her Majesty's Jubilee.

### MORE NURSES FOR INDIA.

The Government in India have requested that six more nurses shall be sent out from England to help in the nursing of the plague patients. The nurses are now being selected by the India Office, and it is hoped they will be ready to start this week or next.

### THE ARMY NURSING SERVICE.

There have been a good many changes in the Army Nursing Service of late. Superintending Sister Thomas has gone from the Station Hospital, Canterbury, to Cambridge Hospital, Aldershot; Superintending Sister Ryan, from Aldershot to Malta, for duty at the Station Hospital, Valletta, relieving Superintending Sister Ferguson, who returns to England after serving five years in Malta. Superintending Sister Burke (Dover) has resigned and leaves the service this month. Sister Holland has been moved to Canterbury from Malta, being succeeded by Sister Wright from Dover. Sister Murray is on her way out to Egypt for duty. Sister Bloomer has been ordered from Aldershot to Dublin, and Sister Ottley from the Curragh Camp to Aldershot. Sisters Milne and Bucher have recently returned to England after five years spent at Gibraltar.

### A GRAND ENTERTAINMENT.

It is announced that some time in June a great and special entertainment will be given at the Lyceum Theatre in aid of the Commemoration Fund for the Queen's Jubilee Institute, the performance to be also something of a "demonstration by the dramatic profession in Her Majesty's honour." Sir Henry Irving and Mr. Edward Terry are concerned in its arrangement, and no pains will be spared to make the whole thing a success.

### A PATIENT'S VIEW.

Some expressions of opinion which have reached us from "grateful patients" should comfort those nurses who have lately felt themselves hardly treated by the public, which hastens to secure their services when ill, and then proceeds to abuse the whole body of nurses with an entire lack of discrimination and much vigour. A recent correspondent, who "has been under the care of hospital nurses and private nurses at home and in nursing homes at various times," writes of "the great kindness and excellent treatment always received at their hands," and indignantly objects to the idea that they should be debarred from theatres and "worldly" amusements. On this latter point all sensible people are of course agreed.

### MIDLAND TRAINING INSTITUTION FOR NURSES.

The Birmingham and Midland Counties Training Institution for Nurses seems to be in a most satisfactory and flourishing condition. There are eighty-nine nurses on the staff, eighty fully trained and nine probationers. The pension fund for the nurses stands at £3,953 10s. 8d., and the sinking fund for providing new premises at £1,516. The committee now have announced their intention of adding another £100 to each of these funds; to make the usual grant to the District Nursing Society, and in commemoration of the Queen's long reign to make a donation to the Society of £200. It has also been decided to divide £200 amongst the nurses and staff in similar commemoration. The Lord Mayor of Birmingham, who presided over the annual meeting, spoke cordially of the "skill, ability, and great kindness of the nurses."

### A SENSIBLE PROPOSAL.

At a recent meeting of the Forden (North Wales) Board of Guardians, when the question of the employment of a district nurse by the Guardians came up, Mr Bircham, the Local Government Inspector, pointed out that boards of guardians were empowered to appoint district nurses where needed, that meaning an outlay of £60 or £70 a year. He added that he always advocated the engagement of a district nurse by the Guardians, a course which would result in great saving to the union.

### NURSING NEWS FROM DUBLIN.

The board of Dr. Steeven's Hospital, Dublin, are proposing to build a new Nurses' Home, apart from the hospital, on an open site commanding a pleasant outlook towards Phœnix Park. This will involve a considerable outlay, but a portion of the money needed has already been promised by generous friends of this old institution, which dates from the days of Queen Anne.—Miss Sarah Hampson, late superintendent of the Rotunda Hospital, opens her private hospital in the premises of the old Portobello Hotel early next month. A well-lighted operating-room has been constructed, with tiled floor, cemented walls, and all modern appliances. Miss Hampson intends providing accommodation at three separate rates of charges, so that the needs of all classes of patients may be met.

### FORFAR AND QUEEN'S NURSES.

AT a recent meeting held at Forfar, under the presidency of the Hon. C. M. Ramsay, it was unanimously decided that the county should commemorate the sixtieth year of the Queen's reign by contributing to the Queen's Jubilee Institute. Forfar has good experience of the value of efficient district nursing. There are 13 Queen's Nurses now at work in the county.

### HULL DISTRICT NURSING ASSOCIATION.

THREE fully-trained nurses are now employed by the Hull District Nursing Association (affiliated to the Q.V.J.I.), and it is hoped the present year may see the much wished for addition of yet another. The fifth annual report, just issued, shows a great increase in the work of late, and the committee earnestly ask for a corresponding development in local support, which they have a right to expect. They suggest a hope that the inhabitants of Hull and the neighbourhood will "make a strenuous effort to help forward the fulfilment of Her Majesty's good wishes for her own nurses by raising a substantial sum to form an endowment for the Hull branch of her institute."

### FOR THE CONSIDERATION OF THE L.G.B.

A STATE of friction has been existing for some time past at the Boston Workhouse between the head nurse and the master and matron. From the evidence revealed at recent meetings of the Board of Guardians it seems probable that there have been faults on both sides, but it is also plain that the nurse is a capable woman, who is admitted to "look carefully after the sick," and that the Board will lose a good and efficient officer if she goes. It is only one more instance of the utter "unworkableness," to coin a word, of workhouse nursing. Were each department properly defined the Boston Board of Guardians, like many another, would not have to waste its time inquiring into petty squabbles which are the inevitable result of the present arrangements, neither would they lose good workers in consequence of the perpetual overlapping of authority which is now the normal condition in smaller workhouses.

### NORTH LONDON NURSING ASSOCIATION.

AT the annual meeting of the North London Association for the Sick Poor the following resolution was passed: "That this meeting recognises the great services which the North London Nursing Association has rendered to the sick poor of the neighbourhood for many years past, and commends it to the hearty support of all residents in the district, and approves of the efforts proposed to be made in this Diamond Jubilee year for obtaining a large increase in the list of subscribers." The Rev. R. M. Thornton, who put it to the meeting, said he was specially struck in reading the report at the extensive character of the work of the association, and the amount of good done at comparatively small cost. A cordial vote of thanks was passed to the superintendent and nurses for their "kind and skilful attention" to their patients, and their general "zeal and devotion."

### THE VISITING NURSE.

THE paper read by Miss Diana C. Kimber at the recent American Superintendents' Convention on "Trained Nursing for People with Moderate Incomes" is published in full in the March number of *The Trained Nurse*, and is well worth reading. It shows how the plan of nursing by the hour, day, or half-day amongst that large class of people who cannot afford the entire services of a first-rate nurse, and are yet by no means objects for charity, has "caught on" in America. Miss Kimber gives the actual experiences of several nurses in the States who have started on these lines, and have found the plan to work well.

### IN THE LAW COURTS.

AN action for damages for libel was recently brought by Miss Fiske, a nurse at the Great Yarmouth Workhouse Infirmary, against a clerk of the name of Palmer, in the employment of the same union. Previous to the action a Local Government Board inquiry resulted in the clerk being censured for his conduct in making the statements he did. Miss Fiske was not satisfied that her character was sufficiently vindicated by this proceeding, and therefore brought the action referred to. Owing to the bad state of Mr. Palmer's health, Miss Fiske consented to accept from him an apology in open Court, and £200 by way of damages, in addition to the legal costs.—The superintendent of the Eastbourne Nursing Association has obtained an injunction against a nurse formerly employed by her, who, contrary to contract, has nursed on her own account in Eastbourne since the termination of her engagement at the institution. The Judge, in summing up, pointed out that it was open to the nurse to bring an action for wrongful dismissal if she held that the superintendent had not good reason for terminating the engagement.

### NURSES ABROAD.

SISTER Sarah Elizabeth Oram, upon whom the Royal Red Cross was recently conferred by the Queen for services to the sick and wounded in the Soudan, received her nursing training at the London Hospital.—Miss Wildman, of the Indian Nursing Service, has not yet fully recovered from her late serious accident. She is still at the Colonial Hospital, Gibraltar, and unable to walk, though we are glad to hear she is gradually recovering her strength.—The Rev. J. Ferrier, whose marriage to Miss Hallowes (daughter of the late Colonel Hallowes), Nursing Sister at the General Hospital, Umballa, we announced the other day, has seen active ervice in his capacity of army chaplain. During the Afghan War, on Lord Roberts' famous march to Kandahar, he was twice mentioned in despatches on account of his devotion to the sick and wounded.

### SHORT ITEMS.

THE recently-started "Flannel Shirt Club" is doing well, and a first distribution of delightful garments has already taken place. Some 150 shirts have been despatched to the medical wards of several London Hospitals, where we are sure they must have been welcomed with enthusaism by the Sisters.—A successful concert was given in aid of the Heywood and District Nursing Association the other day. The programme was a good one and the room well filled by an appreciative audience. The proceeds reached the satisfactory sum of £45 9s.—Is there no district nursing institution or other useful charity needing help in the borough of Godmanchester (Huntingdon) that the form of "Queen's Commemoration" chosen for this year should be the expenditure of £100 on a gold chain for the mayor?—The Pension Fund for the nurses of the City of Dublin Nursing Institution has reached a sum of over £500, though only started towards the end of January.

### Lectures to Surgical Nurses.

By H. A. LATIMER, M.D. (Dunelm), M.R.C.S. of Eng., L.S.A. of London, Consulting Surgeon, Swansea Hospital; President of the Swansea Medical Society; Lecturer and Examiner of the St. John Ambulance Association, &c.

### III.—ATTENDANCE ON THOSE SUFFERING FROM WOUNDS—NECESSITY FOR CLEANLINESS IN SURGICAL DRESSINGS—THE BLOOD; ITS COMPOSITION.

HAVING instructed you as to the methods by which wounds are healed it will be necessary now to tell you what your duties will be as nurses when you are in attendance upon persons who are suffering from such injuries. First and foremost, it cannot be too strongly impressed upon you that in all you do you shall ever bear in mind that the important thing is to be exceedingly cleanly. I dare say you have often wondered, during the course of your training, why you have been required to do many acts of an almost menial character, such as washing and scrubbing waterproof sheetings, and scouring utensils. You may have thought, when feeling tired after ward work, that such duties might well have been left to scrubbers and housemaids, and that they can hardly have formed a part of the necessary training of nurses. But, inasmuch as you have to be trained into habits of excessive cleanliness in small matters, it has been wisely thought that you should yourselves do any kind of work which is a part of the whole duty of a nurse; and the more you are drilled in all details, the more completely are you likely to form habits which will render you thorough in all you do. Disease germs cling with great pertinacity to wounds and to the surroundings of sickness, and, if we are to continue to obtain the splendid results in surgery which mark our present era, it will only be by maintaining the care and attention to all details in operating, and in dressing wounds, which are the main characteristics of modern surgical work.

If a wound has been inflicted purposely, as is the case when a surgeon is operating for the cure of disease, it will be an incised one, and with modern antiseptic or aseptic surgery it is customary to see its edges unite by first intention. You will see, in cases of this sort, the patient prepared for the operation he has to undergo by the parts in the neighbourhood of the disease being carefully washed beforehand, and by their being subsequently rendered free from germs by the careful application of lotions which prevent germs from developing, should they have alighted there. Then, being brought down to the operating-room, you will notice the surgeon and his assistants wash their hands and arms and carefully attend to their nails before they handle the patient or any of the instruments they are about to use. And, further, you will not fail to observe that the most scrupulous care is taken throughout the operation, and from henceforth, by the surgeons and skilled nurses, to ensure a continuance of these cleanly precautions; and you will quickly notice that the success which any surgeon obtains is in proportion to the thoroughness with which all these minute details are carried out by himself and others. As I hope to deal with the subject of aseptic and antiseptic surgery in a later lecture, I will not dwell any longer on this point now, merely saying that I have introduced these remarks to illustrate what I wish to enforce most strongly, viz., that in all you do you shall bear in mind that you are to be constantly waging a warfare against disease germs which, although invisible to you, are, none the less, potent realities. It will not be often that you will have the first dressings to apply to wounds, but it may be that such duties may fall to your lot. Should it chance to be so, remember that the wound must be carefully cleansed of all dirt, and that it must be well irrigated with some antiseptic lotion before it is closed: that its edges will have to be brought together evenly to facilitate the process of healing; and that, if stitches have to be applied to maintain the cut edges in their position, these stitches will have to be tied with just sufficient force to bring the edges into contact, and no more: for tight stitches are very injurious by setting up pain and disturbance in wounds.

Lacerated, contused, and punctured wounds, have all to be dealt with as their several varieties call for. The general principles which have to be borne in mind are: Extreme cleanliness of the wound, of your own hands, and in all instruments used; the free use of antiseptic lotions; and thorough rest to the affected parts. As instruments can be more thoroughly cleansed than hands can be, dressings near a wound should be renewed with forceps, which should have been taken from an antiseptic lotion, and which should be replaced there after having been used.

As the loss of blood from divided vessels necessarily occurs when wounds have been inflicted, we will now consider this subject of bleeding, or hæmorrhage, as it is called. Everybody knows what an important constituent of the body the blood is; how it is the fluid which carries nourishing properties derived from the food we have eaten, and a purifying gas called oxygen, with which we have charged it during the act of breathing, all over the body. I propose to now instruct you as to its composition, the mode by which it is circulated, and the vessels wherein it is contained; and then to show you how to deal with an escape of it from the body, and with the injurious effects upon the system should such an escape have taken place.

Blood, then, is composed of a fluid containing little round cells which are called blood corpuscles. These corpuscles are of two kinds, red and white, and the red cells, which give the red colour to the blood, are vastly in excess of the white ones. By means of this fluid and these cells, foods and a gas are enabled to be taken in, and carried all over the body; and wherever a part which is doing its work healthily requires this food or gas it abstracts the same from the blood, giving up in return worn out materials, and another gas, carbonic acid, to be got rid of by the various excretory channels of the body. The pure gas, oxygen, comes from the air around us. As we fill our lungs with air by an in-going breath, it passes into the blood which is contained in the fine blood vessels which are spread out on the walls of little spaces in those organs; and, passing away from the lungs in these vessels, it is carried all over the body, and, uniting with a material called carbon, there it forms an impure gas called carbonic acid, which in turn comes back to the lungs in other vessels, and is expelled from the blood at the outgoing breath during the act of expiration. Understand: these acts of purification of the blood by gases entering it, and leaving it, at the lungs, go on in a constantly orderly succession. Whilst we are in health and our organs are doing their work properly, we breathe in: oxygen enters the blood; we breathe out: carbonic acid gas leaves the blood.

In order that this fluid shall be conveyed all over the body it has to be kept moving or circulated in the vessels which contain it. The prime moving force is the action of the heart, an organ situated on the left side of the chest, which may be described as a muscular pump, having four chambers.

### Death in our Ranks.

WE regret to announce the death of Nurse Elizabeth Langley, which took place on the 3rd of this month at the Royal Infirmary, Preston, from typhoid fever, contracted in the discharge of her duties. Nurse Langley was only twenty-four, and her loss will be greatly felt by many to whom she had endeared herself.

### Post-Graduate Clinics for Nurses.

By a Trained Nurse.

### VI.—POINTS FOR PRIVATE NURSES.

The views expressed by my American friend in a recent issue of The Hospital have, no doubt, given rise to some flutterings and discussions in private nursing circles; for we "on this side" are hardly prepared to accept in entirety the suggestions she set forth. In the United States that attitude towards her patient which this nurse describes comes more naturally and easily because, as I have previously explained, most American hospitals receive paying patients, and the nurses are consequently, during the whole of their training, inculcated into habits of more "fussy" and pronounced toilet attention than is possible in our large Training Schools where only the poor are treated. And I must confess to having noticed some evils arising from a too decided attitude of personal attendance on the part of a nurse towards her patient; for, after all, though I think a nurse should display the utmost interest in her patient's appearance, and be cheerfully willing to set her or him off to the best advantage, the primary reason for her presence in the sick-room is for the care of the disease, not the individual as an individual. Personal attention is added on, and should be regarded as a legitimate addition, and a necessary consequence of the illness from which the patient is suffering. Were he well he would brush his own hair, and would, doubtless, bestow great pains that it should be as ornamentally and becomingly finished as the nature of the materials at his disposal would allow. But he is ill. Under these circumstances I, his nurse—although that is not the motive of my existence and profession—must cheerfully do for him that which he would much rather do for himself. And I act proxy in my most generous spirit, and fashion his locks as carefully and ornamentally as he with his Bond Street traditions could wish. And because all work well done is a credit, I will put as much art into this simple matter as I would into an intricate piece of surgical bandaging. In every case—or nearly so—the nurse will find that her patient will thoroughly appreciate the spirit of her work, and will harbour no feeling of want of respect towards the profession because of a small detail of this nature. The most dignified persons one knows are, at the same time, the most simple. And dignity is the more easily maintained by those who never think of it. A striving for a dignified effect always places the striver in a most undignified light. Frankly, it has always seemed to me that some American nurses who undertake the manicuring of their patients—the nail polishing, burnishing, &c.—do overstep the limit of that by which we bound the nursing profession. That her nails should be bright and polished is not necessary to the self-respect or dignity of a sick person. It is a step beyond, and borders on the line of artificial "make-up"—and with this the nurse has no part. But here again there is the question of "other countries other customs." An English nurse has not been imbued from childhood with the American view that manicure and nail polishing is a part of every-day "grooming"; and that a nurse should burnish her nails would appear as natural to an American woman as it would to an English patient that her nurse should plait her hair.

There is another point connected with private nursing ethics I have been asked by many nurses to comment on. And that is the question of how far a nurse may recommend foods and sick-room appliances. So many private nurses complain that by their recommendations of certain invalid diets and nursing instruments, &c., they incur a suspicion on the part of their patients that their recommendations are interested. Of course, it often happens that the most disinterested motives are suspected: this being the penalty we must pay for living in an un-Arcadian community. But the nurse is quite safe in her recommendations if the medical man in attendance concur with them. And it is a wise precaution—except in very minor appliances—to consult him on the matter of invalid beds or couches, or any appliance which calls for considerable outlay.

I propose in these clinics to form a kind of Nurses' Parliament, and, to make this more complete, I shall, when important ethics come under discussion, "interview" experienced nurses and give my readers the benefit of their expert views. This week I give the opinions of two very experienced private nurses on the question of "recommendations."

The first one said: "Having been a private nurse in London for so many years, I am fairly well known, and am constantly receiving circular and other letters from quite eminent firms of druggists, instrument and 'dressings' manufacturers, pointing out that they allow so much commission to nurses who recommend their wares. I am also inundated with letters from perfume and soap dealers, toilet and tooth powder patentees, proprietors of baby's soothers and infant clothing specialists, pointing out how my small income may be materially increased by recommending their various wares—all 'of the very best manufacture and eminently satisfactory,' &c. Now, I do not approve of the perquisite system. I would as soon think of taking a 'tip' from a patient's friends. I invariably make it a rule to boycot any firm which suggests a commission to me, keeping a black-list of those who send such propositions. Of course I think that manufacturing firms should decidedly send out circulars to nurses telling of new inventions for the sick-room, and improved contrivances for the comfort of invalids. It is a great advantage to nurses to be kept *au fait* and up-to-date in all such matters. But, at the same time, I maintain that nurses are quite sufficiently honourable and conscientious as to be able to recommend foods and furniture without the obnoxious 'tip' or commission being held out as a bait. But, alas! for the comfort that conscientious persons get for their higher motives—it is the commonest thing where one in all single-mindedness and simplicity advocates the use of any particular patent, for one's patients to suspect at once an underlying interested motive! I often hear nurses say, 'One might just as well get something out of it because people always think we do.' To these I preach the value of *mens conscia recti*—the consciousness that one is acting disinterestedly and honourably."

The next nurse approached is of equal experience, and proved more cynical in her views. She said: "The statement made by many nurses in private practice of the offering by manufacturers and instrument makers of commissions on 'the business they bring' fits in with my own experiences. But I cannot understand nurses being surprised at such offers, for the whole commercial, and even the social, world revolves to the tune of 'you do something for me and I'll do something for you.' 'Backsheesh' is as familiar a cry in London as in the East, only we call it by another name. It is the foundation of society. Take it away and the whole superstructure falls to the ground. I ask my friends to a certain number of dinners in the year. They ask me to exactly the same number at their house. And so on in every branch of life—business, political, and social. And so the world wags." This is very satirical, but, fortunately, not quite true. Anyway, let us hope the world will never so wag that nurses will merge their traditions and ethics in the commercial spirit of to-day. By all means let them, as a part of their duty, endeavour to find out the very best varieties of invalid foods, peptones, appliances, and bedside comforts. But let them choose and recommend these on the highest grounds, and with a single view to the welfare of their patients.

### The Royal National Pension Fund for Nurses.

### THE TENTH ANNUAL GENERAL MEETING.

The tenth annual general meeting of the members of the Royal National Pension Fund for Nurses was held at the offices, 28, Finsbury Pavement, London, E.C., on Thursday, March 11th, 1897. Mr. WALTER H. BURNS, chairman of the Council, presided, and there were, amongst others, present: Mr. Henry C. Burdett, Mr. Gerrard Norman, Mr. Arthur H. A. Morton, Dr. Herbert P, Hawkins, Dr. E. C. Perry, Mr. E. C. Povey, Mr. C. Eric Hambro, Hon. Egremont J. Mills, Mr. George King, F.I.A., F.F.A., and Miss Cave, Miss Nott-Bower, and Miss Monk.

The SECRETARY (Mr. Louis H. M. Dick), having read the notice convening the meeting, the report and balance-sheet, which had already been printed and circulated, were taken as read, on the motion of the Chairman.

### MR. W. H. BURNS, THE CHAIRMAN'S ADDRESS.

Ladies and Gentlemen,—In coming before you for the tenth time, I have very little to add to the annual report which has been laid before you. The year, like the previous one, has not been very eventful, but it is a record of continued prosperity. I will as much as possible continue my observations upon the lines of my last year's remarks in order that the comparison between the two years may be more easy. The proposals received for pension show a slight falling-off as compared with 1895, but a very large increase over the average of previous years, being 244 more than in 1894 and 321 more than in 1893. Premiums received amount to nearly £10,000 more than in 1895.

The total investments, which were £256,111 in 1895 for the pension fund only, now amount, exclusive of cash at the bankers, £8,614, to £311,210; and for the Junius S. Morgan Benevolent Fund they amount to £10,782. The total assets, therefore, at the end of 1896 reached over £330,000. The expenses for the year 1896 amount to £2,266, as against £1,659. To the extent of £87 10s. this increase has been caused by the honorarium which we are now paying to our medical officer, Dr. Potter, but we feel convinced that indirectly his great skill is saving us more than the amount by avoiding losses which would have been otherwise incurred in sickness assurance. The remaining increase is due to the general development of the society's business, as shown in the report. The investments during the year have amounted to over £50,000, and the average return upon them is a trifle over 4½ per cent. I am glad to say that the value of the society's securities at the market prices on December 31st last is considerably above the cost in our books, and this is equally true if the valuation were taken to-day. There are no securities in default.

The number of policies issued during 1896 shows a falling off of only 20, as compared with 1895. This result is very striking, considering that we offered to nurses no special attraction as in 1895, when the Marlborough House presentation drew many nurses into the fund. Consequent upon the increase in the number of policies the amount received in premiums is considerably higher. Our regular premium income may now be considered as at least £50,000 a year. The number of sickness insurance policies taken out shows a large falling off, 272 being issued, against 325 last year. I do not think that there is anything to account for this beyond a mere coincidence, many nurses who joined for pensions not requiring sickness insurance. The amount of the sick pay shows a very large increase—£1,158 was paid in sickness allowance, against £909 in 1895—235 recipients were paid, against 177 in the previous year. Including all cases, the average per head by the recipients has been £4 18s. 7d., against £4 12s. 5d. This includes five chronic cases, who received allowances practically during the whole year.

Two hundred and fifty-three policies were cancelled during 1896, of which practically one half were surrendered on account of marriage, death, and giving up nursing. After allowing for substituted policies, deaths, &c., 238 remain. For these 238 surrendered policies £8,139 6s. 9d. was paid by the Fund against a trifle under £6,000 paid out last year. I find that, in many cases where nurses have withdrawn and given various causes, such as inability to keep up payment, &c., they, as a matter of fact, got married and gave up nursing as a profession. The number of marriages during this year is very remarkable, as in 1895 there were only 29 against 62 in 1896.

The J. S. Morgan Benevolent Fund has been very active in its good work, and our thanks are more than ever due to the ladies of the committee, and to Miss Pritchard and to Miss Leigh. The special appeal which was made to them in connection with this fund to celebrate the sixtieth anniversary of Her Majesty's reign threw a vast amount of extra labour upon the Pension Fund staff, but it is more than gratifying to think how liberally the nurses responded to the good work. Over £2,500 in money and £100 a year in subscriptions were received from them. In acknowledging the gift to the chairman, the Princess of Wales stated that: "The first act of the Prince of Wales on New Year's Day was to present her with the munificient offering from the Nurses of the Royal National Pension Fund to the Junius S. Morgan Benevolent Fund. The Princess feels that the year of the longest reign in English history could not have been more auspiciously inaugurated than by the voluntary contributions of the members of the same association to their sick and suffering sisters." (Hear, hear.)

I am sure that the gracious words of the Princess, and the fact that the first thoughts of the Prince and herself on this eventful year were with her nurses, will prove a precious remembrance to them.

During the present year several members of Mr. Junius S. Morgan's family have supplemented the nurses' donation by an additional equal amount, thus bringing the Junius S. Morgan Benevolent Fund up to £16,000, a sum which the committee think sufficient for the present wants of the nurses.

During the past year we have completed the affiliation scheme with the Consumptive Hospital at Brompton, the Homœopathic Hospital at Birmingham, the Worcester General Infirmary, and the Dublin Trained Nurses' Institute.

The large increase in expenses which you will have noted has been more than justified by the augmented work. As the nurses increased, the number of communications received and sent, and the number of receipts and entries that have to be made are proportionately augmented, and with our small staff are the source of much labour. You can form some idea when I tell you that the number of communications received last year was 26,886, or 2,100 more than in 1895; while the letters posted amounted to 26,575, an increase of 3,000 over the previous year. We issued 15,115 separate receipts, exclusive of the Benevolent Fund, 3,000 more than the previous year, of which 1,206 were to nurses paying premiums in the office, and 1,700 cheques were sent for sick pay alone. We issued 449 armlet badges to the nurses during the year, and I may roughly estimate that the entries that have to be made in our books annually are considerably more than 75,000.

As the Fund grows older, the working out of the surrender values of the policies takes a very large amount of time. Every policy which has been in existence over four years has to be carefully worked out in order to arrive at the precise surrender value in accordance with the terms of the prospectus, and, therefore, I am sure that the members of the Pension Fund will feel that the expenses of the office are not too heavy. I may say that the percentage is 3·74 of our premium income, as compared with 3·42 last year.

The profit on the working of the business and its investments shows that the state of the fund is undoubtedly sound. We have now, apart from the ordinary annuity fund, a bonus fund of £70,000, so that the Pension Fund finds itself in a position of great prosperity, At a former meeting of the nurses at Marlborough House, held some years ago, I ventured to predict that within twenty years from its foundation the accumulated fund of all kinds would reach £1,000,000 sterling, a sum which then seemed to many an extravagant estimate. When I say that at the end of the first ten years' period, which we have now reached, the funds amount to over £330,000, and that we are accumulating at the rate of over £60,000 a year, it seems quite safe to expect that at the end of the next ten years we shall reach the amount which I have already predicted.

I regret to have to announce to you the resignation from the Council of the Hon. Herbert C. Gibbs, owing to pressure of business and inability to attend to the duties of the post. He leaves us bearing the gratitude and goodwill of his colleagues, and I am sure he feels the same lively interest in the Fund which his father and himself have shown hitherto. We have strengthened the Council by the addition of two members—the Hon. Egremont Mills and Mr. Charles W. Trotter—and in concluding my remarks I desire to bear especial tribute to the zeal and devotion of Mr. Dick (our secretary) and his faithful and efficient staff. (Hear, hear.) Our business is prosperous, well organised, and under God's blessing is doing good and useful work. (Applause.)

Mr. BURDETT: I have much pleasure in seconding the adoption of the report. In doing so I think it is only right, as representing the council and also the nurses, to especially thank in your names Mr. Morgan's family for their munificent contributions to the benevolent fund, and also those members of the fund, the nurses, some 700 in number, who spontaneously came forward and raised £2,500 more among their friends, making some £5,000 altogether. Many nurses, in addition, put down their names for subscriptions of 1s. and upwards, and by this means an additional income of £125 per annum was raised. I consider these shillings very precious, both in the history of the fund and in connection with its influence for good. There is one other thing I want to say —and I think it ought to be said—about the benevolent branch of this fund. It does not exist in order to relieve employers from their liabilities to provide adequately for those they ought to safeguard and protect. There is a growfeeling that managers have the right, when their matrons and nurses are members of this fund and come upon the sick fund, to say to them: "Although you have been in our service for years, we shall discontinue your salary as long as you remain ill, and use it to pay a *locum tenens*." The savings which nurses pay into this Fund, for their own protection, are not to be used for the benefit of their employers! We must strongly resist this injustice to our policy-holders, and see that none of the institutions which employ nurses are permitted to trench upon the small savings of the thrift.

There is another point which I think striking. When this Fund was originally started many reasons were urged against it, and perhaps the strongest of all was that the majority of nurses said they did not want a pension fund because they had other prospects in view. Those other prospects were the reasonable and natural hope that some day they would enter "the holy estate." I have always met the objection in this way: "If you are attractive in your present circumstances, you will be doubly attractive with the means for a very handsome trousseau." This fact is borne out by the figures in the Chairman's report, for whereas the number of marriages in 1895 was only twenty-nine, they amounted to sixty-two last year, or had more than doubled. I think it is a matter of which we may be proud that this Fund should have been the means adopted by the Prince and Princess of Wales to commemorate in their own way, as the first of Her Majesty's subjects, this memorable year. I know that the Princess of Wales takes a great interest in this Fund, and her gratification was more than shared in by the Prince when they knew of the handsome additions to the Fund made by Mr. Morgan's friends and by the nurses themselves. (Hear, hear.) I have very much pleasure in seconding the adoption of the report.

The resolution adopting the report, accounts, and balance sheet was carried unanimously.

### ANNUITANTS' REPRESENTATIVES.

The CHAIRMAN: The next business is the ballot for the annuitants' representatives, the result of which will be declared by the scrutineers.

Mr. GEORGE KING read the following report: "We hereby certify that we have examined the ballot cards received for the voting for the annuitants and policyholders' representatives. One thousand three hundred and seventy cards were received; two were spoilt, or contained more than seven names. The following is the result of the voting: Miss Mabel Cave, 1,304 votes; Miss Mary L. E. Dunn, 1,318; Miss E. Fisher, 1,316; Miss L. M. Gordon, 1,330; Miss K. H. Monk, 1,317; Miss F. C. Nott Bower, 1,312; Miss E Vincent, 1,315; Miss M. Shirley, 24; one lady, 3 votes; two ladies, 2 votes; nineteen ladies, 1 vote. We are informed that 3,821 cards were sent out. Seeing that this year it was necessary for the policy-holders to pay the postage, we think that the fact that over one-third took part in the voting shows that the policy-holders take a great interest in the result.—(Signed) GEORGE KING, GEO. W. POTTER." Miss Cave, Miss Dunn, Miss Fisher, Miss Gordon, Miss Monk, Miss Nott Bower, and Miss Vincent were accordingly declared elected.

The retiring members of the Council—Mr. John Watney, Mr. G. Norman, Dr. E. C. Perry, Mr. A. C. Rothschild, and Mr. Thomas Bryant, F.R.C.S.—were unanimously re-elected.

### RE-ELECTION OF AUDITOR.

Mr. MORTON: I have been asked to propose the re-election of Mr. Whinney as auditor, and have very great pleasure in doing so.

Mr. NORMAN seconded, and the re-election was unanimous.

The CHAIRMAN: The Council has decided to recommend that the auditor's fee should be fixed at thirty-five guineas. Hitherto it has been twenty guineas, but the auditor reports that owing to the enormous increase of work this sum does not even cover the cost of clerical labour.

The increase of the fee to thirty-five guineas was agreed to unanimously.

### VOTE OF THANKS TO THE CHAIRMAN.

Mr. BURDETT: I have the pleasure to propose a vote of thanks to the chairman. It has been my pleasure to do this for many succeeding years, and I hope it may be my privilege to do it for many years more. Mr. Burns is the life and soul of this Fund, and it is a great thing that we should have a man of his position to give time and thought to our institution. I think he has given in his speech the best evidence of the value of his services to the Fund in the statement of the position of the funds. It is a great thing to be able to say that we have no single security under cost value, and that i all the securities were realised to-day we should make a handsome profit on them. Personally, like other members of the Council, I regard Mr. Burns, not only as a colleague, but as a dear and good friend, too. In many ways he helps forward the fund, and is always ready to conserve its best interests. It is evident that so long as he remains chairman, it will continue to prosper. I have very great pleasure, therefore, in proposing this vote of thanks to Mr. Burns.

Dr. Perry, who seconded, said: The functions of a medical man on the Council do not clash with those of an important man like Mr. Burns, and we are now not often called upon to give our opinion even on a medical question, owing to the very great care exercised by Dr. Potter in the duties which he so ably carries out. (Hear, hear.) No one can sit at the Council Board and fail to see that Mr. Burns is the backbone of the institution. While we are not without other helpers, I suppose every impartial observer will admit that his services are very great in a pecuniary sense, and in time and brains. (Hear, hear.)

The Chairman: I have had to return thanks eight or ten times for the tribute you have given me eight or ten years, and I am very deeply touched by the remarks of the proposer and seconder. If I have done my best, I have been more than ably seconded by the colleagues whose help has never failed. Mr. Burdett, as the founder of the Fund, is entitled to much more credit than I am. We have been working in perfect harmony for ten years with perfect success, and the nurses are well pleased. I was very much struck with Mr. King's remarks.* After hearing him, it is quite clear that we are beyond possible competition, and if we do make a profit it all goes back to the nurses in bonuses. If they can get better terms elsewhere we only wish they may. We have only their interest at heart, for nobody offers more hearty and willing service. Gentlemen, I thank you for your kindness. (Applause.)

The proceedings then terminated.

* These are unavoidably held over until next week.

### Appointments.

### MATRONS.

Workhouse Sick Wards, Hampstead.—Miss Jane Jones has been appointed Night Charge Nurse at this workhouse. Miss Jones received her training at Fir Vale Infirmary, Sheffield, and has, since leaving that institution, held the post of charge nurse at the Toxteth Park Infirmary, Liverpool, and at Gore Farm, and the Northern and Western Fever Hospitals of the Metropolitan Asylums Board.

### Presentations.

At a meeting of the Hospital Committee, held at the Ladywell Sanatorium at Eccles (County Borough of Salford), on February 22nd last, the chairman of the Health Committee, Mr. Councillor Huddart, presented to Miss Thomas, the lady superintendent, on behalf of the committee, an illuminated address with a purse containing 20 guineas. The address was signed by the Mayor of Salford, the ex-Mayor (Sir W. H. Bailey), the Chairman, Deputy-Chairman, and all the members of the Health Committee, as well as by the Medical Superintendent and the Medical Officer of Health for the Borough, as a mark of their appreciation of Miss Thomas's valuable and faithful services for over twenty-one years in that capacity, and of the esteem in which she is held by all the members of the committee.

### Nursing in America.

The fourth Annual Convention of Superintendents of Training Schools for Nurses met in Baltimore on February 10th, when nearly fifty regular members of the society were present, and several visitors. The papers, though few in number and short, were valuable and interesting. Important statistical work is being done by the society, and the summing up of the results shown by the collection of data from schools all over the country, gathered with infinite pains by Miss Walker in her paper on "The Progress of the Three Years' Course," and by Miss McKechnie on "What has been accomplished in the Direction of Uniformity of Teaching," was, on the whole, highly satisfactory. Great interest was shown in the paper on the "Brooklyn Associated Alumnæ Registry," by Miss Merritt, while Miss Kimber's paper on "Trained Nursing for People of Moderate Incomes," and Mrs. Robb's paper on "The Nursing of Small Hospitals," were heard with interest and approbation. In the discussion which followed the latter, the President of the Milwaukee Training School gave an instructive account of the co-operative work done by that school, which at present is doing the nursing in seven small institutions.

The President elected for the coming year is Miss Snively, and the next meeting will take place in Toronto in February, 1898. The other officers are, Miss Nutting, vice-president; Miss Drown, treasurer; Miss Dock, secretary; Miss Brennan and Miss Stowe, auditors; and Miss Palmer and Miss Nevins, councillors.

Two delightful receptions were given in honour of the Society, one at the Johns Hopkins Hospital, by the Trustees and Officers of the Hospital, and one by Mrs. William Osler; hospitality was also shown in other special honours, among which were the opening of the doors of the Arundel Club, of which Miss Elizabeth King is President, to the society; the opportunity given by Mrs. Bonaparte to visit the historical relics of the Bonaparte House; and the unusual privilege granted by Mr. Walters of a special opening of the famous Walters Gallery.

The second meeting of the Convention called a year ago by the American Society of Superintendents of Training Schools for Nurses, for the purpose of organising an American Association of graduate nurses, took place in Baltimore on February 11th and 12th, 1897.

The first meeting was held at Manhattan Beach last September, when a Constitution and By-laws were drafted, providing for an association which should cover the United States and Canada. At the Baltimore meeting, just held, the work of the Convention was completed, the Constitution and By-laws, as drafted, were considered, altered in various details, and formally adopted by the delegates in the name of their respective Alumnæ Associations. The officers of the new organisation were elected, and the first meeting voted to be held in New York in about one year's time.

Mrs. Robb, formerly superintendent of the Training School of the Johns Hopkins Hospital, in whose mind the thought of a national organisation of nurses had taken form many years ago, and with whom its consummation has ever since been a leading purpose, was elected President. The two Vice-Presidents, who will at present have direct charge of the work of associating the various Alumnæ—the one in this country, the other in Canada—are Miss Lena A. Walden, of the New York Hospital, and Miss Ambros, of the Presbyterian Hospital, New York. The Secretary is Miss Barnard, of the Johns Hopkins, and the Treasurer, Miss Tamar Healy, of the Brooklyn City Alumnæ. A number of Alumnæ delegates were present, and delegates from the Society of Superintendents. The meetings were held in the Nurses' Home of the Johns Hopkins Hospital, where the delegates were hospitably entertained at luncheon on both days.

### A Royal Visit.

### THE ALEXANDRA HOSPITAL FOR CHILDREN WITH HIP DISEASE.

A BRIGHT and cheery sight greeted us in the matron's sitting-room at the little hospital in Queen Square when we dropped in one dull afternoon. Round the fire was clustered a group of little ones, looking like a bunch of poppies, clad in their scarlet jackets and grouped close together, so as not to over-crowd the small domain. Tea with matron was just over, and there was a busy hum of happy little voices. "The hospital had just had a great honour," wrote one of the girls to the friends at home, and the honour was a visit from the Princess of Wales! Many years ago the Princess gladly consented that so admirable an institution should bear her name, and so the hospital for children suffering from hip disease has always been known as the Alexandra, and just before our visit the Princess had signified her intention of coming to see "her" little ones, without any formality. She arrived, accompanied by Princess Victoria and Miss Knollys, and, after a brief visit to matron, she said, "Shall we go and see the children," and, accompanied by the large collection of toys, one for each little patient, the Royal party mounted the steep and narrow stairs. At the entrance of the first ward stood the oldest inhabitant—Willy Martin—promoted to crutches, and in the place of honour to receive Her Royal Highness. Much impressed with the dignity of the position was Willy, and when we asked him what he had done with the Royal lady's present he replied, with much solemnity, "Mother's got it at home with a label tied on 'to Willy Martin from the Princess of Wales.'" Visiting day had taken place shortly after the Princess's visit, and the treasured toys were sent home to be carefully guarded as lifelong possessions, probably labelled after the manner of Willy Martin's.

With her own hands the Princess had presented her gifts to each little sufferer, gaining the admiration of the elders present by her thoughtful selection, and anxiety that each child should have what it desired. She was quite distressed to find that she might have added to the variety of gifts by the introduction of some paint-boxes, which she knew were not admitted into most hospitals, but which find their way like many another special favour into this happy refuge for little sufferers. The Princess expressed great pleasure when she saw rosy cheeks amongst the pale ones, and her pity was greatly provoked for the tiny mites lying so patiently there in the far from comfort-suggesting attitude necessary in the treatment of the disease. The children, however, fortunately become so accustomed to straps and position that they are quite amused if discomfort is suggested.

One omission was regretfully mentioned by the nurses after the visit, and this was the special introduction of "Jubilee Jones" to Her Royal Highness. The Princess talked some time beside his bed, but she was not made aware of his historic identity, which, doubtless, would have interested her, as it is not everyone who can claim the honour of having been born in the famous year after which he was named, only exceeded in importance to English subjects by the present. Perhaps next time Her Royal Highness visits the wards a Commemoration Brown will take the place of Jubilee Jones, by then restored, we will hope, to his place in the world.

One little boy caused some amusement by his strongly-expressed desire to shake hands with the Princess, a request she smiling complied with. The Princess Victoria showed no less interest in the children than the Princess of Wales, and she was much amused by two little people—one of whom, having been given a tea-set, invited her to drink a cup of tea, and who pointed out to her that she had omitted to provide herself with a spoon; and the other, quite a mite of a girl, who, it was explained to her, presented a button-hole every week to her favourite doctor. One day the flowers were forgotten, and when the usual presentation time came round the would-be donor was found bitterly weeping.

Not only did every little patient receive a gift and kindly words from the Princess of Wales, but none of the staff were passed without kindly recognition, and the nurses record with admiration how Her Royal Highness noticed everything. The flowers on the tables, the clean boards—and, alas! their patches—the meagre space, low and narrow doors, and the many deficiencies which made her exclaim, with sympathetic conviction, "You do, indeed, need to build a new hospital," no doubt recognising with admiration the courageous and cheerful spirit of all who work with such success in spite of difficulties and drawbacks which would daunt were not a true love of the work ever present, a fact which no one visiting the Alexandra Hospital can doubt. All the harder is it that just opposite is the beautifully-equipped hospital for paralysis, where the necessity for cheering surroundings and space has been fully realised for patients who, like those also at the Alexandra Hospital, are doomed to spend months, and even years, with no other sights than the hospital ward can afford them. Yet no one has taken the unpresuming little hospital in hand and said this should not remain unaltered another day. With the children in Queen's Square the little institution is not a hospital, it is a home, and for the time—sometimes a very long one—their world also. In all London there is no other shelter where they can be sure of a welcome with the best facilities for recovery, for the other hospitals seldom accept these long and tedious cases, and so were it not for the Alexandra Hospital many a child would languish crippled and in pain in miserable surroundings, improperly nourished, uncared for, and too often unwanted. Perhaps this year of grace may bring the much desired new hospital. The hearts of givers have never been more ready to respond to the calls made upon them, and who knows but the kindly visit of Alexandra, Princess of Wales, to the hospital which is named after her, may not result in kind friends coming forward and securing for it another visit from her very soon, when she will be able to say not "You do indeed need new buildings," but "Now I see you have all that you want."

### Minor Appointments.

NICHOLSON MACKENZIE MEMORIAL HOSPITAL, STRATHPEFFER.—Miss Mary Grassick has been appointed to the Matronship of this hospital. Miss Grassick was trained at the Aberdeen Royal Infirmary, and has since been engaged in private nursing.

CANCER HOSPITAL, STANLEY GROVE, MANCHESTER.—Miss Nellie Spirey has been appointed Sister at this hospital. Miss Spirey was trained at the Royal Albert Edward Infirmary, Wigan; she was afterwards appointed sister there, and subsequently held a similar position at the Wirral Children's Hospital, Birkenhead.

BRITISH LYING-IN HOSPITAL, ENDELL STREET, W.C.—Miss Ada F. Rockett has been appointed Assistant Matron at this hospital, the appointment dating from April 15th next. Miss Rockett was trained at the Leeds General Infirmary and at Queen Charlotte's Lying-in Hospital, and holds the L.O.S. certificate.

ROYAL ALBERT HOSPITAL, DEVONPORT.—Miss Annie Santo has been appointed Sister of children's wards at this hospital. Miss Santo was trained at the Royal Albert Hospital, where she was considered "an excellent nurse." Subsequently she has worked at the hospital, Burton-on-Trent, as charge nurse, and at the South-Eastern Fever Hospital, New Cross, which post she resigns to return as sister to her former training school. Miss Santo holds good testimonials.

### Real Nurses.

Lady Priestley's article on nurses of the present day has caused a large amount of comment. Unfortunately much that she depicts is true of a part of the nursing profession, but it would be a grave mistake to think that in the present day the public cannot safeguard itself from the employment of unsuitable nurses. We cannot do better than draw their attention to the aims of the Nursing Sisters of St. John the Divine. We have heard so much in praise of the nurses trained under their system that we quote certain paragraphs from their present report, which in a great measure explain the reason of their success:—

"And now we come to an aspect of the question that modern freedom of thought seems to wish to thrust aside, or at best, to leave alone. We mean the motive for which, and for which only, the work is done. We are accused of having thrown away all idea of a religious element as a factor in the work, not to say as forming its principal source of strength, that we work simply for a livelihood, that we have forgotten the message that has come down to us through the ages, 'Inasmuch as ye did it unto the least of these My brethren, ye did it unto Me.' That the Roman Catholic Sisters do better than we, because they have remembered this, and that it has stood to them in place of training and knowledge. This they do not themselves allow, many of the Irish nuns, the 'filles Dieu' of the sister isle, engaged in hospital and infirmary nursing, having begged for more training, that they may be enabled to do better work. But even if it were the case, it must be remembered where the responsibility for it rests. The pressure put upon all English nurses who wish to take their place in the first ranks of their profession has been—and is—enormous. A curriculum is put forth, through which they must pass (or be refused the desired certificate), which would be most trying even if they were not at the same time going through the severe and protracted physical strain which a hospital training entails. Everything possible is done to ensure their technical knowledge, but mostly little to train their hearts and minds. And those who have for some years done their utmost to promote this state of things should hesitate to censure a condition of affairs which they have themselves created and fostered, and should not condemn hastily those nurses who have accepted too literally the only standard apparently held up to them.

"We have tried always to impress upon our own nurses that these aims are not high enough—that it is safest to 'hitch their wagons to a star;' and we feel we must be right, for letter after letter comes to us from the patients they have nursed and from the friends of those who cannot speak for themselves—the aged or the very young, the dying and the dead—all repeating the same kind and encouraging words, telling of the comfort and help the nurses have been, of their kindness, care, and patience, and that they are a support and not a trial in the homes in which they find themselves domesticated.

"The public cannot judge fairly the hardships and trials sometimes the fate of the 'private nurse.' She sees so often the seamy side of human life. The patient, in his misery, forgets the courtesies, nay, sometimes even the very decencies of life. The friends, too, may be inconsiderate, and make no effort for her comfort; or there may be no friends, and she must face it all alone. And to stand all this—far more than to remain simple and unaffected in the midst of the 'luxuries and indulgences' showered down upon the favourite nurse—she requires to be armed with the strongest principles and the highest guides of conduct, and for the one, as well as for the other, must be taught and encouraged to set her face towards righteousness. The rest will surely follow."

### St. Luke's Home, Vancouver.

(From a Correspondent.)

Once more we have a year's work to report, and thankfully are able to say that it has been, on the whole, a good year. Financially things have been fairly satisfactory. We have no further indebtedness to report, though we would gladly get rid of the burden of the eight per cent. interest on the old debt on the building of £500.

Our present staff consists of Sister Frances (in charge), Nurse Jeannie, Nurse Margaret, and Nurse Alice. The total number of patients nursed has been sixty-eight; thirty-seven were nursed in the home, the others at their own homes. Vancouver is going ahead, it is all the gold mines now. Lots of hospitals are cropping up everywhere; all the little Canadian and Pacific Railway stations have one, and there are some good openings for nurses in these new places. One of our nurses has settled at Nelson, where she found enough work to send for her sister to help her, and both are doing well. Mr. Clinton is still here, working as hard as ever, but we have lost Dr. Bell Irving. He has gone in for mining. Dr. Johnston is here still.

We had a very quiet Christmas owing to two serious accidents being brought in to us about that time; one an English lady who was thrown from a buggy while driving to a friend's house in the country among the mountains. She had to be left on the road unconscious while assistance was got, and has been three months recovering. The other case was that of a clergyman who was found twenty feet below the railway, fallen, it is supposed, from the train. At first he was thought to be injured fatally, and was probably only saved by the snow, which was deep where he fell. He was discovered quite by accident about four hours after quite unconscious, and though he is now almost himself again he can remember nothing prior to the accident of his life and surroundings. With these two anxious cases we kept our festival very quietly.

Amongst the visitors to St. Luke's Home during the past year may be mentioned the Bishop of Quebec, the Rev. J. C. Roper (of Toronto), Dr. Louisa Clarke and Dr. Landis (from Corea), the Misses Wright, and the Rev. Mr. Mackay (from Australia). It is with great pleasure we feel that the home has been the means of helping some few, at any rate, and that its work is likely to increase in the future.

### Where to Go.

Royal British Nurses' Association.—The fourth sessional lecture of the season will be given on Friday, March 26th, 1897, at 8 p.m., at the offices of the Royal British Nurses' Association, 17, Old Cavendish Street, W., by F. J. Wethered, Esq., M.D., F.R.C.P., the subject being "Bacteriology," with demonstrations. Members are admitted free, the general public on payment of 1s.

The Victoria Commemoration Club, 29, Southampton Street, Strand, W.C.—Mr. G. Drummond Robinson, M.D., will deliver a lecture on "Antiseptics in Midwifery Practice" at this club on Tuesday, March 23rd, 1897, at 7 p.m. Tickets may be obtained from the secretary; 1s. each to members, 1s. 6d. to non-members. If to be sent by post, a stamped envelope must accompany the application.—Miss Earle, of the National Training School of Cookery, will begin a course of practical demonstrations on "Dainty Dishes for the Sick-room" at the club on Tuesday, March 30th, at 7 p.m. Tickets for the course of six lessons may be obtained from the secretary; price 5s. to members, 7s. 6d. to non-members. Application for tickets must be made before March 30th.

Miss Molony gave a very interesting lecture on "The Training of Masseuses" at this club on Tuesday last, in which she touched upon the mutual advantages that might follow if hospitals would allow masseuses free entry to the wards to masse patients for whom this treatment might be considered desirable. All masseuses are willing to give their services for the experience thus gained in handling helpless subjects. During her training a masseuse deals only with a healthy model, who can move and turn as directed, and it is not until she is a qualified masseuse and practising that she comes across difficult heart cases and often totally helpless cases. If the hospitals would accept this suggestion the patient's convalescence might be hastened, while the practice and experience afforded to the masseuse would be considerable.

### A Book and its Story.

"BENJAMIN'S SACK."

Miss Meta Scott's story* opens with a scene at a man's death bed. The man dies, and we hear no more about him, but on his dying wish the plot of the story is more or less based. This concerned the marriage of the deceased's daughter, of whose destined husband he said, "The lad has promise of turning out a brave Catholic gentleman, and my girl—her dead mother's memory bears out—will make no worse wife for being Protestant. . . . Cousin Matilda, if she keeps the land, be he your son, or any son, so long as he be of the faith which has ever ruled here, the girl must marry a Catholic. . . . Send me in the girl and the lad. I would see the little pair before I go."

Ten years afterwards Agnes Angèle and Adolphe Seymour, the girl and the lad destined for one another, were man and woman, and Cousin Matilda had watched them grow to such.

It was a recognised fact in the parish of Muthallen that Agnes Angèle was born into the world for the sole purpose of uniting the two branches of the Muthallen family by marriage with her distant cousin, Adolphe Seymour. The Angèles of Muthallen owned all the straggling parish, and much of the fertile valley in which the hall lay, which also embraced the hills that surrounded it—low-lying spurs of the Cheviots, that here and there struck out to sea.

The Muthallen natives, loyal to the traditions of long descent, with which they associated the family, boasted of its antiquity that when "The first Angèle of Muthallen died, it was then that the race of angels in heaven began."

The family, as the name testifies, were of Norman extraction; "their name, their land, and their Roman Catholicism were the three things they had always held inviolable."

Agnes' life had been an uneventful one; her quiet, retired existence had known no experiences beyond the confines of the little sea-board parish where her lot was cast.

Her education had been conducted by the local clergyman, and between the tutor and his pupil a great friendship existed. Benjamin Stone was poor, he lived in a frugal manner at a simple home, his sister keeping house for him. The devotion of this sister, Margaret, was strong, with a curiously misdirected zeal of its own; a love that set aside all scruples of all other considerations—as we shall see.

Now, Benjamin Stone had written a book; and all that lay in the way of publishing the said book was the necessary wherewithal. To bring it out, the firm, to whom he had submitted it for approval, had offered terms which required an outlay of £50 on the author's part; this sum, paltry as it might seem, which stood in the way of the writer's future success, was not forthcoming—and Benjamin Stone had not the means of furthering his claims to literary immortality. And here it is that his sister's devotion displayed itself. With all the uncontrolled love which a woman at bay is capable of, Margaret Stone displayed an heroic, though misdirected zeal on her brother's behalf. If by fair means the necessary money for the publication was not forthcoming, then the woman insisted to herself it must be by means other than fair. We are first introduced to brother and sister, when the means have been found—Margaret Stone hands the money over to her brother, answering him no questions as to its source, on which she is silent.

Now the truth was, that to obtain this money the vicar's sister had carried out a robbery; and though she pacified her conscience as best she could, and sought to justify the end by the means, the woman was guilty of deliberate theft. It was Agnes Angèle's money she had taken—"Agnes Angèle, whose coffers were so full"—she may have pleaded to herself in extenuation of her conduct, and yet Agnes Angèle was a lifelong friend—a young girl who had trusted her. For an account of the method employed for the extraction of the bank notes, the reader must be referred to Miss Scott's own words. Suffice it here to say, the deed was done, and the young girl was not only conscious of the betrayal, but of the perpetrator of the deed. It did not take long for her to grasp the reason of this daring deed on Margaret Stone's part, and, having grasped the reason, Agnes, with a ready sympathy and immutibility uncommon in woman, guarded her friend's secret, and, moreover, forgave her. Agnes' loyalty to her friend distinguished itself in the manner with which she withholds from Margaret Stone knowledge of the fact she is in possession of the secret. The deed done, the writer portrays in a dramatic manner the effect produced on the woman's mind—remorse and contrition, combatting with love of her brother, a love which set alike at defiance all laws and all considerations.

We do not remember to have seen Miss Scott's name very frequently in literature, and yet her analysis is by no means the work of an immature thinker or writer.

About this time Adolphe Seymour makes a discovery, and traces the theft of his cousin's money to the Vicarage, ultimately attaching the blame on the worthy Vicar. On this subject a scene ensues between the two cousins. Agnes insists on his silence; he, with all the fiery impetuosity of his nature, demands the right to proclaim the offender; for the time being, the girl's gentle insistence quiets him. The young man's passion is further augmented when he discovers that more than friendship is felt for his cousin by the guilty vicar—a Protestant into the bargain—to whom, overcome with rage and indignation, Adolphe Seymour explains his position, that he is in possession of the secret, and any provocation on the vicar's part will cause him to expose this to the world.

Benjamin Stone was an honourable man, his position not an enviable one if he bore the blame of the deed; the Church he represented would be defamed, and yet Benjamin Stone never wavered about this—his sister must not be betrayed. The plot works on, and though full of incidents, we must keep to a bare outline of the story. Suffice it to say, Margaret Stone dies, and when she dies it is Agnes Angèle who ministers to her last hours. The woman died unconscious of the consequences of her deed, supported by the knowledge that she had managed to return the stolen money.

And what his sister's love had caused her to do, the expenditure of a zeal which was meant for his doing, but which in reality was his undoing, the clergyman of Muthallen bore the full weight of; and finally, a disgraced and dishonoured man, he left the little parish which he had loved. The wish of the dying man at the beginning of the book was not fulfilled, for when we last see Benjamin Stone and Agnes Angèle it is no longer in their former relations of master and pupil, but in the nearest of all human relationship, and we leave the two strong in a bond of love which defied all obstacles, having made their heaven out of the ruins of the past. The theme of the story may be a sad one, it is never morbid, but healthy and stimulating, and we see two characters which are not subjugated, but strengthened by sorrow and mischance, whose lives come together in the end in a way which would not have happened had the roads been smooth ones on which they trod.

* "Benjamin's Sack." By Meta C. Scott. (Ward, Lock, and Bowden, Limited. London. 1896.)

### Wants and Workers.

Nurse M. M., 31, Pullen Road, Boxmoor, Hemel Hempstead, Herts., will be grateful for any old sheets or underlinen for a serious case of typhoid fever in a very poor family. Full particulars will be given if required.

### Everybody's Opinion.

[Correspondence on all subjects is invited, but we cannot in any way be responsible for the opinions expressed by our correspondents. No communication can be entertained if the name and address of the correspondent is not given, or unless one side of the paper only is written on.]

### CYCLING FOR NURSES.

A "Nurse Cyclist" writes: Being an old district nurse, and having used a cycle for several years, I have come to the conclusion that cycles are excellent for long distances when the weather is suitable and the roads good, but that a district nurse cannot count on being able to use a cycle every day in the week, and certainly not every day in the year. On country roads during a wet or snowy winter she may not be able to use it often during December, January, and February. Windy, wet, or dusty days I prefer walking twenty or more miles to labouring over heavy roads or against the wind. Cycling is also more trying than walking during the hottest part of the day of the hottest months. Usually in the very hot time I use mine early in the morning and after half-past four p.m. You also require someone to clean and put your cycle in order each day. If constantly used the repairs amount to a considerable sum in the year. I do not think a cycle a profitable investment for a district nurse for her work, though it is exceedingly convenient and pleasurable for occasional use. I use no cloak, simply a uniform dress, sleeves, and apron, bonnet, and a divided skirt to ankles under dress-skirt made of the same material. I am a devoted cyclist and should not now like to think of life without one, but I like good roads and good weather for it.

### LADY PRIESTLEY AND THE NURSES.

"Sister Ruth" writes: I should like to say a little on the unfair and unjust attack on nurses by Lady Priestley. I certainly think that Lady Priestley should have trained in hospital for a nurse, and then have taken up private nursing for at least three months, before she commenced such an attack on nurses. By that time she would have been better able to write about them; she evidently has never had the privilege of meeting a real nurse, one who has taken up the work (not as a new road to matrimony), but as a conscientious worker among the sick and suffering. For the last five years I have been in and out among all classes of patients—male and female, rich, middle-class, and poor—as a private nurse, and I can with truth say that, in almost every case, patients and their friends have been very grateful for help I have given during the time of suffering and anxiety; and among my truest friends are my old patients and their relatives. Of course, I have had my difficulties and trials (with patients' friends more particularly), and it is very difficult to keep clear of unpleasantness in many houses; but if the work is done with a right motive, and the relatives are allowed free intercourse with the patient, much of the suspicion of, and dislike to, a nurse is soon done away with, and in almost every instance that I have had to keep the friends out of the room, they have been very nice about it, when the reason for doing so has been explained to them. I think that when such attacks are made on a body of women whose life and work is one long self-sacrifice, it can only be done by those who are utterly ignorant of the mind of a true nurse, and the real desire of doing all in her power to restore the sick one to health. I fear Lady Priestley has met some who are only nurses in name, women not fully trained, and for their very unfitness were sent away from the hospital, and these are women whom we real nurses do not acknowledge as belonging to our class. I would like to say also that a real nurse could not possibly work for less than "two guineas" a week, because she would need rest between cases, and a summer holiday, and I am sure the wear and tear of our life is well worth such a fee, or more. As to places of amusement and recreation, I certainly think it is good both for patient and nurse, for after a little recreation of that sort the nurse is brighter, and can interest and amuse the patient by little details of what she has seen and heard; and, moreover, the patient is very often very much interested and delighted by these little anecdotes, and able for a time at least to forget pain and weariness. I would suggest that her ladyship should undertake to nurse properly a bad case (say of pneumonia), beginning work at seven p.m., staying up all night, and keeping on duty until two p.m., when she would get a few hours' rest; be up and on duty again at nine p.m., and stay until two p.m. next day; and so on each day until the patient was convalescent. I think by that time she would feel glad to get out to a concert or matinée, or even a game of tennis; and she would also learn in that time that a private nurse's life is not quite what she thinks it is, and I am sure she would have more respect for nurses as a body. Failing this, the next best thing would be for her to need nursing herself, and be in the hands of true nurses, who would return good for evil, and so help her to retract her tirade. One thing I am thankful for is that, with one exception, I have never nursed anyone with such ideas of nurses as Lady Priestley has.

### A VISIT TO THE VICTORIA COMMEMORATION CLUB.

Mrs. Braidwood, formerly lady superintendent of the Wirrall Children's Hospital, writes: There are many nurses, some distance from London, who are wishing, I am sure, that they could run in and see this new nurses' club that is just started close to the Strand. I wanted much to see it myself, and as I had to meet a friend in town I thought what a pleasant thing it would be to see her at the club and have our private talk in comfort there. She was on her way to a case, had about two hours to spare in London, so was glad enough to join me there. We arrived in Southampton Street and quickly found the handsome new building—The Hospital Building—in which the Nurses' Club is most appropriately situated. The courteous secretary, to whom we made known our wants, showed us the rooms of the club. And, oh, nurses, what delightful, charming rooms they are. The drawing-room, always warm, and lighted by electric light, is most tastefully arranged. The colours harmonised well, and the Egyptian rugs and ornaments give it quite a unique appearance, which is a restful change after the monotony of the wards, and the hideous rooms one has often to live in. But comfort is not sacrificed to appearance. The couches and easy chairs are substantial enough for the heaviest of us, and so cosy. In the next room, furnished, as they all are, in equally good taste, are writing tables lighted with special electric lights and amply provided with club writing paper and every other necessary. All nursing papers, as well as others, are provided for the use of members in these rooms. Besides convenient lavatories, there is a good-sized toilet or cloak-room, furnished with hooks for cloaks, &c. On the table stands a box that is filled with plenty of pins of all kinds, including hair-pins and hat-pins; a really nice place to tidy one's self after being in these rough March winds. I must tell you, too, that we had our lunch there. Such dainty little menu cards are placed on the table, and the plates and dishes, and in fact everything is so exceedingly pretty, it makes one feel quite hungry. Evidently the secretary knows well the wants of a nurse when she is away from her work, and sympathises with her need of pretty things to see and use sometimes. I found that for one shilling I could attend the first of the lectures held in the club lecture-room. After my friend left me I went up into the room and took my seat. The room was full, and no wonder, for what nurse is there who is not anxious to increase her stock of knowledge? The lecture, which was upon the chemistry of food, and was very interesting and useful, was given by Mrs. Alting Mees. I hear that the secretary is planning out the lectures for us, and the next will be given in the charming little club kitchen I expect, for it is a demonstration by one of the experts from the South Kensington School of Cookery, and will teach us how to make "small dainties for the invalid." For those who do not already know, I must add that the member's subscription for the use of these lovely club-rooms is only one guinea per annum, and there is no entrance fee until after June. It is intended to make it a sort of commemoration scheme for the benefit of nurses. The rooms are enough, and large enough to accommodate a good many; and it is to be hoped that many will join, for the expense of keeping it all going must, of course, be great. The only thing charged for is food, and the charges are so exceedingly moderate that no member can grudge the small cost It is about the same as the charges at an A.B.C., but the surroundings are so luxurious and the cups and saucers simply sweet. I think that nurses who do not expect to use the rooms much might become members for the benefit of other nurses, for the more the club flourishes the more will the clever and energetic secretary do for the ncreased comfort of the nurses. I did not see any accommodation for the nurses' bicycles, but no doubt that will be provided as the need arises. Do not wait for that, however, before you join, but see or write to the secretary and secure your membership before the numbers are filled up.

A CORRECTION.

A PARAGRAPH in the *Practitioner* suggests that the "associated workers" who are eligible for membership include "young doctors and medical students." It need hardly be said that this is entirely incorrect. The club is exclusively for ladies, but, as at all such clubs, gentlemen are admitted as visitors when accompanied by a member.

### For Reading to the Sick.

"INWARD TRUTH."

**Verses.**

LIE not; but let your heart be true to God,
Thy mouth to it, thy actions to them both;
Cowards tell lies and those that fear the rod;
The stormie-working soul spits lies and froth,
Dare to be true. Nothing can need a lie:
A fault, which needs it most, grows two thereby.
—*George Herbert.*

Words are mighty, words are living;
Serpents with their venomous stings,
Or bright angels crowding round us;
With heaven's light upon their wings;
Every word has its own spirit,
True or false, that never dies,
Every word man's lips have uttered
Echoes in God's skies.—*A. A. Proctor.*

Think truly, and thy thoughts
Shall the world's famine feed;
Speak truly, and each word of thine
Shall be a faithful seed;
Live truly, and thy life shall be
A great and noble creed.—*Bonar.*

This above all, to thine own self be true,
And it will follow, as the night the day,
Thou can'st not then be false to any man.—*Shakespeare.*

Take all in a word: the truth in God's breast
Lies trace for trace upon ours impressed;
Though He is so bright and we so dim,
We are made in His image to witness Him;
And were no eye in us to tell—
Instructed by our inner sense—
The light of heaven from the dark of hell,
That Light would want its evidence. —*Browning.*

**Reading.**

It is of vast importance whether the soul, which is to live for ever, is a truthful, pure, and noble soul, made strong through the conquest of many and great temptations; with affections set upon all that is good and beautiful; with conscience that clearly sees the difference between right and wrong, and a firm will, resolute to choose the right.—*Martineau.*

I wish to be alive to all the little low and dark motives which are continually coming into the soul, and which, I believe, if they are not marked and continually carried up to a higher power to be prayed away, are ever liable to settle there, and thence to come out in some questionable and deceitful action. Truth, real inward truth, is the rarest, I think, of all things. Some little petty subterfuge, some verbal or acted dishonesty, we are continually surprised into; and against this neither a high code of honour nor an exact profession of religion is much preservation. Continued intercourse with the Father of Light, revealing our own darkness in us, is, I am quite sure, the one safeguard, and a Christian who should lose this is in more danger of stumbling than an infidel.—*F. D. Maurice.*

Falsehood is so easy, truth so difficult . . . examine your words well and you will find that even when you have no motive to be false, it is a very hard thing to say the exact truth, even about your own immediate feelings—much harder than to say something about them which is not the exact truth.—*George Eliot.*

### Notes and Queries.

*Midwifery Training.*

(171) At what cost could I get experience in midwifery in a district, and lectures from a doctor, in order to pass the L.O.S. examination?—*Enquirer.*

The Secretary of the Midwives' Institute, 12, Buckingham Street, Strand, can recommend suitable district midwives who take pupils, and they have courses of lectures at the institute on purpose for midwives' pupils going up for the L.O.S. See note on this subject in "THE HOSPITAL" NURSING MIRROR for March 13th. The cost is about 16 guineas inclusive.

*Physical Training.*

(172) Can you tell me anything about Madame Oesterburg's Physical Training College, or any other colleges or gymnasiums for remedial calisthenic exercises?—*Erica.*

Write to Miss Dove at the Society of Trained Masseuses, 12, Buckingham Street, Strand, W.C. Doubtless she will be able to give you information on this subject.

*The Hospital Commissariat.*

(173) Please tell me what weight of tea is usually allowed for each patient per day in most hospitals, ¼ or ½ an ounce?—*Sister Alice.*

The usual allowance of tea is a pint twice a day, and one ounce will make six pints. The estimate would therefore be one-third of an ounce per day. Read the articles which have recently been appearing in THE HOSPITAL on "Hospital Expenditure: The Commissariat."

*Sanitary Inspectorships.*

(174) Can you tell me anything about the training for these, and at what age I could begin to work up for such an appointment?—*Hygiene.*

See reply to similar query in this column in the "Mirror" for March 6th.

*Army Nursing Service.*

(175) Can you tell me if three years' training at a provincial hospital of 72 beds would qualify for appointment on the Indian Nursing Service or the Army Nursing Service?—*E. C. S.*

Write for particulars to the Director-General of the Army Medical Department, War Office, Pall Mall, S.W.; and to the India Office, St. James' Park, S.W.

*Nursing the Lepers.*

(176) Please tell me where the Leper Island is, and give me information about going out and nursing the patients?—*Nurse Valetta.*

If you mean Robben Island, it is opposite Table Bay, Cape Colony. If you look back through your HOSPITALS you will find an account of the island. There is no demand for nurses to go out there.

*Children's Nurses.*

(177) Kindly tell me the address of the institute where girls can get some months' training to fit them for the post of children's nurse in a private family?—*Sister Matron.*

The Norland Institute, 29, Holland Park Avenue, W.

*Army Nursing Uniform.*

(178) Where can I get the regulation collars and cuffs worn by the Army Nursing Sisters?—*H. Y.*

Write to the Superintending Sister at Netley Hospital. Harvey and Nicholl, Knightsbridge, supply the regulation uniform to the Indian Nursing sisters, and have all patterns.

*Nurses in 1896.*

(179) Kindly tell me in which numbers of THE HOSPITAL of last year I shall find the rules, &c., for the nurses of the Middlesex and West London Hospitals?—*J. B.*

The article on the Middlesex Hospital appeared in THE HOSPITAL for August 8th, and that on the West London in the number for November 7th. You can get the back numbers from THE HOSPITAL office by writing to the manager.

*Information Wanted.*

(180) Please advise me (1) as to the best means of training for district nursing, and obtaining such work; (2) are there any general hospitals where a young woman, 23½ years of age, could train for a year, receiving salary from the beginning; and (3) would previous practice as a dispenser affect the question of remuneration at all?—*S. B. R.*

(1) Read "District Nursing," by Amy Hughes, and "How to Become a Nurse," published by The Scientific Press, 28 & 29, Southampton Street, Strand, W.C. In the latter you will find a list of London and provincial hospitals, with particulars of the training offered to probationers. Only a few of the smaller provincial institutions give so short a period of training, and in most cases you would have to pay for anything under two years' training. If you want to become a nurse why not go in for it thoroughly and enter some good general hospital for at least two years? That you could do and receive a small salary and uniform from the beginning. (3) Please explain what you mean by "practice as a dispenser."

*London and Provincial Hospitals.*

(181) Do you know of, or publish, a list of all the hospitals in London and the provinces, with the number of beds, &c.? If so please tell me the price.—*G. B.*

"Hospitals and Charities," published by the Scientific Press, 28 and 29, Southampton Street, Strand, W.C., price 5s., gives you every information as to hospitals and kindred institutions, not only in London and the country, but all over the world.

*Puro Tea.*

Several correspondents have asked where "Puro" may be obtained. The address will be found on page 418 of this week's issue.

